# Detection of Infectious Poxvirus Particles

**DOI:** 10.3201/eid1207.060093

**Published:** 2006-07

**Authors:** Andreas Nitsche, Daniel Stern, Heinz Ellerbrok, Georg Pauli

**Affiliations:** * Robert Koch-Institut, Berlin, Germany

**Keywords:** Orthopoxvirus, real-time PCR, cell culture, virus replication, diagnostics, dispatch

## Abstract

To enable rapid and reliable detection of poxviruses in clinical and environmental specimens, a diagnostic approach was developed to detect <3 PFU of infectious poxvirus particles in <5 hours. This approach involved virus culture combined with real-time reverse transcription–polymerase chain reaction detection of 2 viral genes expressed immediately after infection.

After the attacks with anthrax spores in the fall of 2001 in the United States, the potential abuse of variola virus or genetically engineered orthopoxviruses in bioterrorist plots has been intensely discussed (*1**–**3*). To date, several diagnostic assays have been developed to rapidly and reliably detect poxvirus particles or poxvirus genomes in suspected samples. Electron microscopy (EM) can also identify poxvirus particles (*4**,**5*). However, it cannot differentiate between orthopoxvirus species and has limited sensitivity because reliable detection is only possible with particle concentrations >10^6^/mL (*6*).

Molecular methods such as real-time polymerase chain reaction (PCR) are more sensitive, detecting <10 genome equivalents per PCR, but PCR can only identify short stretches of poxvirus DNA (*1**,**7*). Nevertheless, since EM and PCR cannot discriminate between infectious and noninfectious virus particles or nucleic acids, they are not satisfactory when an evaluation of the infectious capacity of viral particles is required.

Identifying viral particles by EM is usually sufficient to diagnose a poxvirus infection in clinical samples from patients with typical symptoms of this infection. Virus concentration should exceed 10^6^ particles/mL; however, even at these concentrations only the virus family can be determined, and no additional classification is possible. Detection of poxvirus nucleic acids is sensitive and permits identification of virus-specific sequences and differentiation of a variola virus infection from an infection with other orthopoxviruses. Thus, a combination of both methods is recommended for frontline diagnostic procedures, and a positive result obtained by 1 of these methods would initiate a confirmation diagnosis.

If symptoms in clinical cases are unambiguous, they can usually be attributed to a replication-competent infectious virus. In contrast, in environmental samples, including samples from suspected parcels, a positive EM or PCR result would also require virus isolation to prove that particles could replicate to make a reasonable risk assessment (German Smallpox Preparedness Plan, available from http://www.rki.de).

With environmental samples, the unknown factor is to what extent the sample matrix influences the ability of the virus to replicate, and detecting particles by EM or DNA by PCR does not necessarily indicate infectious particles. The only diagnostic approach to identify replication-competent poxvirus particles is their propagation in a suitable cell culture system. With this system, it takes >1 day to reliably detect poxvirus proteins with specific antibodies.

We combined a cell culture approach that identifies virus replication with the speed and sensitivity of real-time PCR. To this end, we changed the target of real-time PCR from poxvirus DNA to poxvirus mRNA genes that are highly expressed during the first few hours of the infection cycle. Expression levels of these genes enable sensitive detection 1–2 hours after infection. The complete diagnostic approach can be performed in 96-well plates and provides results within 5 hours of receipt of a sample.

## The Study

Briefly, 1.5 × 10^4^ HEpG2 cells were infected with 150 PFU of vaccinia virus strain Lister Elstree. A 15-minute centrifugation step at 1,000 × *g* increased the efficacy of infection by a factor of 10 compared with regular infection at 37°C (data not shown). Virus-containing supernatant was removed, and virus was allowed to replicate for 4 h. Every 30 minutes an aliquot of cells was harvested, and RNA and DNA were isolated by standard procedures (RNAeasy kit and Blood DNA kit, Qiagen, Hilden, Germany). RNA was subjected to 1-step real-time reverse transcription–polymerase chain reaction (RT-PCR) (QuantiTect Probe RT-PCR kit, Qiagen) in a real-time PCR 7700/7900/7500 sequence detection system (Applied Biosystems, Foster City, CA, USA). Amplification of fragments of the *F1L* gene, an apoptosis modulator, and the *rpo*18 gene, the small subunit of viral RNA polymerase (*1*) (both genes are encoded by all poxviruses including variola virus), was monitored by gene-specific 5´-nuclease probes.

Expression of the *F1L* and *rpo*18 genes could be detected 30 minutes and 1 hour after infection, respectively. The copy number of the transcripts was determined by comparison with in vitro translated RNA molecules that were generated according to standard procedures. Briefly, RNA was transcribed in vitro by T7 RNA polymerase (RiboMax RNA production system, Promega, Madison, WI, USA) from plasmids containing the respective PCR target region, and plasmid DNA was digested with DNase.

**Figure 1:**
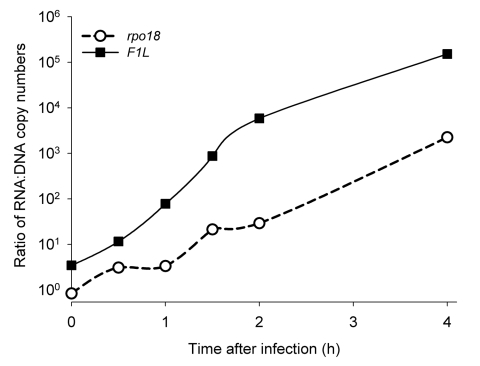
Comparison of RNA load and DNA load of the poxvirus *F1L* and *rpo*18 genes during the first 4 hours after infection with vaccinia virus strain Lister Elstree (multiplicity of infection 0.01). Cells were infected, and at the indicated time points RNA and DNA were prepared and quantified by real-time polymerase chain reaction. The ratio of RNA to DNA molecules is shown.

During the first 4 hours after infection *F1L* mRNA increased 2.7 × 10^4^-fold, indicating early expression of viral genes in the cells analyzed. The *rpo*18 mRNA showed a 410-fold increase after 4 hours. Quantification of viral DNA showed a slight decrease in DNA during the same period, and the ratios of RNA to DNA increased substantially, as shown in Figure 1. This high ratio of poxvirus RNA to poxvirus DNA demonstrates that a possible background of genomic viral DNA, which is derived from poxvirus particles that are noninfectious or from traces of poxvirus genomic DNA in the RNA preparation, does not result in false-positive results in real-time RT-PCR.

**Figure 2:**
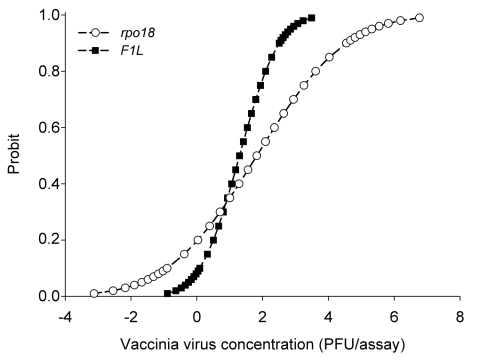
Comparison of probit (predicted proportion of replicates positive) regression curves for *rpo*18 (circles) and *F1L* (squares) genes calculated with SPSS software (SPSS Inc., Chicago, IL, USA). Probit versus vaccinia virus concentration was obtained from 12 replicates of 7 dilutions from 1,500 PFU to 0.1 PFU.

To evaluate the detection limit of our approach, a probit analysis was performed by repetition of the detection (N = 12) of vaccinia virus strain Lister Elstree. Vaccinia virus stocks were titrated according to standard procedures. The virus load used varied from 1.5 × 10^3^ PFU to 0.1 PFU, which is equivalent to a multiplicity of infection of 0.15 to 1 × 10^-5^ (*8*). As shown in Figure 2, after 2 hours of incubation, real-time PCR analysis showed that the *F1L* assay detected 3 PFU of vaccinia virus, and the *rpo*18 detected 6 PFU of vaccinia virus with a confidence interval of 95%.

## Conclusions

The extremely low detection limit of the new assay indicates that environmental samples, which may contain cell culture inhibitory substances and are routinely subjected to crude separation steps such as low-speed centrifugation before analyses, can be diluted by several orders of magnitude to dilute inhibitors while maintaining the viral load at detectable levels. The time frame required for the individual steps of the diagnostic approach is 15 minutes for sample infection, 2–4 hours for virus propagation, 30 minutes for RNA preparation, and 2 hours for real-time RT-PCR. Use of alternative, more rapid real-time PCR platforms further reduces the time required to complete an assay. For poxvirus-positive results, fluorescence melting curve analysis of the *rpo*18 PCR product allows rapid and reliable differentiation of variola virus (*1*). Under optimal conditions, results can be obtained <5 hours after the sample has arrived in the laboratory.

In summary, the combination of cell culture and real-time RT-PCR detection of early, highly expressed viral genes permits detection of minute quantities of infectious poxvirus particles in a suspected sample. Identification of variola virus can be performed by fluorescence melting curve analysis, therefore permitting a reliable risk assessment of a suspect parcel.

## References

[1] Nitsche A, Ellerbrok H, Pauli G. Detection of orthopoxvirus DNA by real-time PCR and identification of variola virus DNA by melting analysis. J Clin Microbiol. 2004;42:1207–13. 10.1128/JCM.42.3.1207-1213.2004 15004077 PMC356842

[2] Whitley RJ. Smallpox: a potential agent of bioterrorism. Antiviral Res. 2003;57:7–12. 10.1016/S0166-3542(02)00195-X 12615298

[3] Tegnell A, Wahren B, Elgh F. Smallpox—eradicated, but a growing terror threat. Clin Microbiol Infect. 2002;8:504–9. 10.1046/j.1469-0691.2002.00525.x 12197872

[4] Niedrig M, Schmitz H, Becker S, Gunther S, ter Meulen J, Meyer H, First international quality assurance study on the rapid detection of viral agents of bioterrorism. J Clin Microbiol. 2004;42:1753–5. 10.1128/JCM.42.4.1753-1755.2004 15071040 PMC387573

[5] Hazelton PR, Gelderblom HR. Electron microscopy for rapid diagnosis of infectious agents in emergent situations. Emerg Infect Dis. 2003;9:294–303. 12643823 10.3201/eid0903.020327 PMC2958539

[6] Biel SS, Nitsche A, Kurth A, Siegert W, Ozel M, Gelderblom HR. Detection of human polyomaviruses in urine from bone marrow transplant patients: comparison of electron microscopy with PCR. Clin Chem. 2004;50:306–12. 10.1373/clinchem.2003.024539 14684621

[7] Olson VA, Laue T, Laker MT, Babkin IV, Drosten C, Shchelkunov SN, Real-time PCR system for detection of orthopoxviruses and simultaneous identification of smallpox virus. J Clin Microbiol. 2004;42:1940–6. 10.1128/JCM.42.5.1940-1946.2004 15131152 PMC404623

[8] Smieja M, Mahony JB, Goldsmith CH, Chong S, Petrich A, Chernesky M, Replicate PCR testing and probit analysis for detection and quantitation of *Chlamydia pneumoniae* in clinical specimens. J Clin Microbiol. 2001;39:1796–801. 10.1128/JCM.39.5.1796-1801.2001 11325993 PMC88028

